# Automated machine learning for early prediction of acute kidney injury in acute pancreatitis

**DOI:** 10.1186/s12911-024-02414-5

**Published:** 2024-01-11

**Authors:** Rufa Zhang, Minyue Yin, Anqi Jiang, Shihou Zhang, Xiaodan Xu, Luojie Liu

**Affiliations:** 1 Department of Gastroenterology, Changshu Hospital Affiliated to Soochow University, Changshu NO.1 People’s Hospital, No. 1 Shuyuan Street, 215500 Suzhou, Jiangsu China; 2 Department of Gastroenterology, The First Affiliated Hospital of Soochow University, Suzhou, Jiangsu China

**Keywords:** Automated machine learning, Acute kidney injury, Acute pancreatitis, Creatinine, International normalized ratio, Predictive models

## Abstract

**Background:**

Acute kidney injury (AKI) represents a frequent and grave complication associated with acute pancreatitis (AP), substantially elevating both mortality rates and the financial burden of hospitalization. The aim of our study is to construct a predictive model utilizing automated machine learning (AutoML) algorithms for the early prediction of AKI in patients with AP.

**Methods:**

We retrospectively analyzed patients who were diagnosed with AP in our hospital from January 2017 to December 2021. These patients were randomly allocated into a training set and a validation set at a ratio of 7:3. To develop predictive models for each set, we employed the least absolute shrinkage and selection operator (LASSO) algorithm along with AutoML. A nomogram was developed based on multivariate logistic regression analysis outcomes. The model’s efficacy was assessed using receiver operating characteristic (ROC) curves, calibration curves, and decision curve analysis (DCA). Additionally, the performance of the model constructed via AutoML was evaluated using decision curve analysis (DCA), feature importance, SHapley Additive exPlanations (SHAP) plots, and locally interpretable model-agnostic explanations (LIME).

**Results:**

This study incorporated a total of 437 patients who met the inclusion criteria. Out of these, 313 were assigned to the training cohort and 124 to the validation cohort. In the training and validation cohorts, AKI occurred in 68 (21.7%) and 29(23.4%) patients, respectively. Comparative analysis revealed that the AutoML models exhibited enhanced performance over traditional logistic regression (LR). Furthermore, the deep learning (DL) model demonstrated superior predictive accuracy, evidenced by an area under the ROC curve of 0.963 in the training set and 0.830 in the validation set, surpassing other comparative models. The key variables identified as significant in the DL model within the training dataset included creatinine (Cr), urea (Urea), international normalized ratio (INR), etiology, smoking, alanine aminotransferase (ALT), hypertension, prothrombin time (PT), lactate dehydrogenase (LDH), and diabetes.

**Conclusion:**

The AutoML model, utilizing DL algorithm, offers considerable clinical significance in the early detection of AKI among patients with AP.

**Supplementary Information:**

The online version contains supplementary material available at 10.1186/s12911-024-02414-5.

## Introduction

Acute pancreatitis (AP) is a common disease characterized by inflammation of the pancreas, affecting adjacent local and peripancreatic tissues. Globally, it has a morbidity rate of 34 per 100,000 individuals [1]. And, cases are distributed without significant differences across age groups and genders [2, 3]. While the majority of individuals with AP experience spontaneous resolution of symptoms, around 20% are at risk of developing severe complications, such as systemic inflammatory response syndrome (SIRS) and persistent organ failure [4]. Acute kidney injury (AKI) is acknowledged as a common complication in AP cases, with its incidence ranging between 10 and 42%, particularly among critically ill patients [5, 6]. Moreover, the prognosis for AP patients who develop AKI is considerably worse, with mortality rates varying from 25 to 75%, accompanied by substantial healthcare expenditures [7, 8]. A study in the United States disclosed that the annual hospitalization costs for AKI exceeded $5.4 billion, ranking it as the second most expensive condition in the U.S. healthcare system, only surpassed by sepsis, which costs about $7.7 billion [9]. A significant portion of these expenses is attributed to cases of less severe AKI [10]. Additionally, the mildest form of AKI approximately doubles the mortality risk, which further escalates to 3–10 times in stage 2 and 3 AKI [11]. The intrinsic risks associated with AP can exacerbate these outcomes. Therefore, early and precise identification, coupled with timely intervention for AKI in AP patients, is crucial.

Previous studies in this field have predominantly utilized traditional regression methods to construct prediction models [12–14]. In these studies, they incorporated biological markers such as interleukin-6, serum cystatin C (CysC), and other variables, making it unfavorable for the model’s generalization and clinical application. Furthermore, these studies typically lacked comprehensive evaluation metrics such as receiver operating characteristic (ROC) curves, calibration curves, and decision curve analysis (DCA) to assess both model performance and clinical applicability.

In recent years, the utilization of machine learning (ML) in the field of medicine, including both supervised and unsupervised approaches, is gaining popularity due to its ability to leverage large clinical datasets and efficient algorithms. In comparison to traditional logistic regression (LR), ML offers notable advantages in predicting complications and other relevant aspects [15, 16]. In the realm of ML models pertinent to this field, while some demonstrated commendable performance, there was a notable increase in complexity. This complexity arose from the incorporation of variables such as serum cystatin C (CysC), intra-abdominal pressure (IAP), and the Acute Physiology and Chronic Health Evaluation II (APACHE II) score. This complexity made them less practical for clinical application and posed challenges in achieving early AKI prediction [17–19]. Conventional machine learning encompasses algorithms such as Support Vector Machines (SVM), Random Forests, and similar approaches. However, a novel form of machine learning known as automated machine learning (AutoML) has emerged, which can intelligently choose from a range of algorithms and hyperparameters to tailor a model specifically for the target data. In comparison to traditional machine learning, the use of intelligent techniques like early stopping, cross-validation, regularization, and hyperparameter optimization significantly reduces the time required to develop more precise models. And, in our previous research on predicting severe acute pancreatitis (SAP), the algorithm performed well, with the best model achieving a test set area under the curve (AUC) of 0.945 [20].

In this study, we utilized routine serological indicators within 24 h of admission for patients with AP as variables. We employed the H2O AutoML platform to train and validate a series of ML models for early prediction of AKI in AP patients. Additionally, we compared their predictive performance with the traditional logistic regression (LR) method.

## Materials & methods

### Patients

From January 2017 to December 2021, a retrospective analysis was conducted at Changshu Hospital Affiliated to Soochow University. The patients were randomly allocated into a training group and a validation group at a ratio of 7:3. As a county hospital, Changshu Hospital has established five major centers, including the Chest Pain Center, Stroke Center, Atrial Fibrillation Center, and so on.

The diagnostic criteria for AP were established according to the revised 2012 Atlanta classification [21]. To confirm a diagnosis of AP, patients needed to satisfy at least two of the following three criteria: (1) experiencing typical abdominal pain; (2) having serum amylase levels surpassing three times the upper limit of normal; and (3) displaying imaging evidence demonstrating characteristic AP findings [21]. According to the the definition of kidney disease: Improving Global Outcomes (KDIGO) guidelines, AKI could be defined in one of the following situations: (1) an increase in serum creatinine (SCr) of 0.3 mg/dl (≥ 26.5µmol/L) within 48 h; (2) known or presumed kidney damage occurring within 7 days, with SCr rising to more than 1.5 times the baseline value; (3) urine output < 0.5 ml/(kg.h) for a continuous period of 6 h [22]. Adults aged over 18 years who met the above criteria would be enrolled in this study. Patients who met the diagnostic criteria for AKI during their entire hospitalization for AP were classified as belonging to the AKI group, whereas those who do not met the criteria were classified as non-AKI group. Patients with chronic liver disease, chronic kidney disease, hematological disorders, recurrent/chronic/traumatic/idiopathic pancreatitis, pancreatic cancer, history of pancreatic resection, those who underwent chemoradiotherapy, and pregnant patients were excluded from the study. All patients received treatment following the guidelines for managing AP. This study was approved by the ethics committee of Changshu Hospital Affiliated to Soochow University (L202324).

## Data collection

Electronic medical records were used to extract demographic characteristics, clinical information, and information on concomitant diseases. These parameters mainly included the patient’s gender, age, etiology, history of hypertension, history of diabetes, systolic blood pressure, diastolic blood pressure, and mean arterial pressure (diastolic pressure + 1/3 of pulse pressure).And, the etiological diagnosis of AP could be primarily determined as follows: for biliary acute pancreatitis, the diagnosis primarily depended on comprehensive inpatient examinations such as abdominal ultrasound, magnetic resonance imaging (MRI), and computed tomography (CT) scans to determine the presence of gallstones and signs of infection in the biliary system, for hyperlipidemic acute pancreatitis, the key factor was whether the serum triglyceride(TG) levels at the time of onset exceed 11.3 mmol/L, for alcoholic acute pancreatitis, the primary consideration was whether there is a clear history of heavy alcohol consumption before the onset of the disease. If the condition did not fall into any of the above three categories, it was attributed to idiopathic acute pancreatitis.

**Fig. 1:**
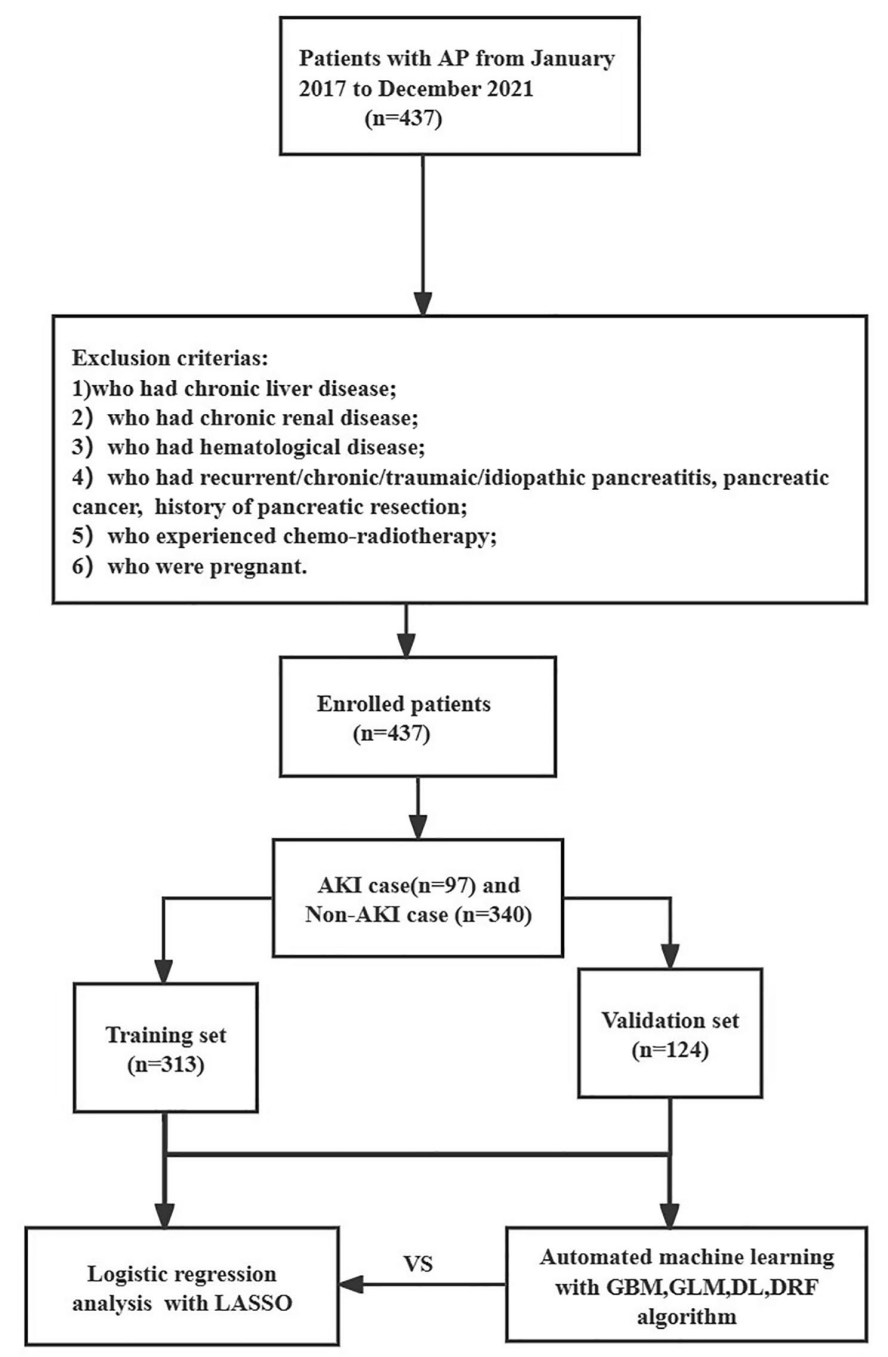
The flowchart of this study

Laboratory data, including blood routine examination, coagulation tests, and serum biochemical tests, were retrospectively collected within the initial 24 h after admission. These parameters included platelet count(PLT), white blood cell count(WBC), neutrophil count(N), lymphocyte count(L), hematocrit(HCT), red cell volume distribution width(RDW), lymphocyte percentage(Lr), creatinine(Cr), total bilirubin(TB), direct bilirubin(DB), urea(Urea), lactate dehydrogenase(LDH), serum calcium(Ca^2+^), triglycerides(TG), glucose(GLU), alanine aminotransferase(ALT), aspartate aminotransferase(AST), gamma-glutamyl transpeptidase(GGT), alkaline phosphatase(ALP), albumin(ALB), amylase(AMY), sodium(Na^+^), serum potassium(K^+^ ), prothrombin time(PT), international normalized ratio(INR), activated partial thromboplastin time(APTT), and C-reactive protein(CRP). In total, 36 variables were analyzed, as described in Supplementary Table S1. Any missing variables were recognized as missing data at random and were imputed using a random forest algorithm via the “mice” package in R software [23]. A flowchart depicting the study is presented in Fig. 1.

### Statistical analysis

If continuous variables followed a normal distribution, they were presented as mean ± standard deviation (SD), whereas if they did not follow a normal distribution, they were presented as median (interquartile range). Categorical variables were presented as frequencies. For the comparison of two groups, categorical variables were analyzed using Pearson’s Chi-square test or Fisher’s exact test, while continuous variables were analyzed using Student’s t-test or nonparametric Mann-Whitney U test. A *p*-value less than 0.05 (two-sided) was considered statistically significant. All statistical analyses were conducted using R software (version 4.2.1) and the following packages: H2O (version 3.36.0.2), tableone (version 0.12.0), tidyverse (version 1.3.0), tidyquant (version 1.0.2), and lime (version 0.5.1).

### Logistic regression algorithms and automated machine learning algorithms

The least absolute shrinkage and selection operator (LASSO) regression model with “λmin” as the criterion was employed for conducting univariate analysis to address the issue of multicollinearity among variables. Utilizing binary logistic backward stepwise regression analysis, we identified independent risk factors. And, these factors were employed to develop a nomogram. The predictive performance of the model was evaluated by ROC curve, calibration curve, and DCA.

The machine learning approach employed the H2O package (www.h2o.ai) for AutoML, which automatically selects appropriate algorithms and incorporates them into ensemble models. This includes the default Random Forest (DRF), a randomized grid of Gradient Boosting Machines (GBMs), an Extremely Randomized Forest (XRF), a randomized array of Deep Neural Nets (DLs), and a predefined array of Generalized Linear Models (GLMs). Hyperparameter optimization involved a 5-fold cross-validation grid search on the training set, assessing various hyperparameter combinations based on their performance measured by the area under the curve (AUCs). Visualizations such as feature importance, SHapley Additive exPlanation (SHAP) plots, and Local Interpretable Model Agnostic Explanation (LIME) were used for presenting the results. Feature importance quantified the impact of each feature on the machine learning model’s outcome. SHAP provided an explanation of the influence of each feature on individual predictions, offering more intuitive and interpretable model explanations. LIME analysis illustrated the contribution of each feature to the prediction outcome by randomly providing examples from the validation set.

## Results

### Baseline characteristics

We included a total of 499 patients in our study, of which 62(12.4%) were excluded due to incomplete data recording. The detailed clinical characteristics and baseline data of 437 patients are presented in Table 1. In the training dataset, males accounted for 56.2% (176/313), while females accounted for 43.8% (137/313). The median age in the non-AKI group was 52 years (interquartile range 40–65 years), whereas in the AKI group, the median age was 54 years (interquartile range 42–72 years). In the validation dataset, males accounted for 55.6% (69/124), while females accounted for 44.4% (55/124). The median age in the non-AKI group was 52 years (interquartile range 38-65.5 years), whereas in the AKI group, the median age was 58 years (interquartile range 44–71 years). Biliary obstruction or gallstones (42.3%) were the most common etiology of AP in our cohort, consistent with previous research. No statistically significant differences were observed in gender, age, smoking history, hypertension history, and diabetes history between the two groups in both datasets (*p* > 0.05).

**Table 1:** Baseline Characteristics of patients

Variables		The training, dataset (*n* = 313)				The validation dataset (*n* = 124)		
	Group	Non-AKI(*n* = 245)	AKI(*n* = 68)	***p***-value		Non-AKI(*n* = 95)	AKI(*n* = 29)	***p***-value
Sex (%)	Male	136 (55.5)	40 (58.8)	0.727		54 (56.8)	15 (51.7)	0.786
	Female	109 (44.5)	28 (41.2)			41 (43.2)	14 (48.3)	
Age(mean (median [IQR])		52.00 [40.00, 65.00]	54.00 [42.00, 72.00]	0.242		52.00 [38.00, 65.50]	58.00 [44.00, 71.00]	0.093
Etiology (%)	Biliary	110 (44.9)	25 (36.8)	0.317		37 (38.9)	13 (44.8)	0.443
	Hyperlipidemia	47 (19.2)	20 (29.4)			14 (14.7)	6 (20.7)	
	Alcoholic	13 (5.3)	3 (4.4)			6 (6.3)	0 (0.0)	
	Others	75 (30.6)	20 (29.4)			38 (40.0)	10 (34.5)	
Smoke (%)	No	221 (90.2)	57 (83.8)	0.208		87 (91.6)	24 (82.8)	0.255
	Yes	24 (9.8)	11 (16.2)			7 (7.4)	5 (17.2)	
Hypertension (%)	No	170 (69.4)	33 (48.5)	0.002		70 (73.7)	18 (62.1)	0.331
	Yes	75 (30.6)	35 (51.5)			25 (26.3)	11 (37.9)	
Diabetes (%)	No	225 (91.8)	62 (91.2)	1		85 (89.5)	27 (93.1)	0.826
	Yes	20 (8.2)	6 (8.8)			10 (10.5)	2 (6.9)	
SBP (median [IQR])		130.00 [119.00, 142.00]	135.50 [120.00, 146.00]	0.073		133.00 [120.00, 143.50]	130.00 [126.00, 151.00]	0.392
DBP (median [IQR])		80.00 [71.00, 87.00]	79.50 [70.00, 88.50]	0.918		80.00 [72.50, 87.00]	80.00 [78.00, 85.00]	0.638
MAP (median [IQR])		96.67 [88.33, 105.00]	96.67 [90.00, 109.50]	0.586		96.33 [89.50, 105.00]	96.67 [94.67, 104.67]	0.313
PLT (*10^9^/L) (median [IQR])		186.00 [146.00, 229.00]	181.50 [132.00, 239.00]	0.684		179.00 [152.50, 225.00]	155.00 [132.00, 214.00]	0.175
WBC (*10^9^/L) (median [IQR])		11.10 [8.80, 14.10]	13.00 [9.60, 16.22]	0.038		11.60 [8.70, 15.30]	10.00 [8.50, 15.30]	0.662
N (*10^9^/L) (median [IQR])		9.10 [6.40, 11.80]	10.55 [6.77, 13.95]	0.055		9.60 [6.25, 12.15]	8.70 [6.10, 13.60]	0.894
L (*10^9^/L) (median [IQR])		1.30 [0.90, 1.80]	1.30 [0.80, 1.95]	0.946		1.20 [0.90, 1.90]	1.40 [0.70, 1.70]	0.456
HCT (L/L) (median [IQR])		0.43 [0.39, 0.46]	0.41 [0.36, 0.47]	0.256		0.42 [0.39, 0.46]	0.41 [0.39, 0.44]	0.064
RDW (%) (median [IQR])		12.80 [12.30, 13.40]	12.80 [12.50, 13.30]	0.971		12.70 [12.35, 13.30]	13.00 [12.60, 13.70]	0.136
Lr (%) (median [IQR])		12.10 [7.30, 17.90]	9.80 [7.07, 15.38]	0.166		11.30 [7.30, 19.85]	11.40 [5.00, 17.50]	0.41
Cr (µmol/L) (median [IQR])		63.00 [53.00, 74.00]	72.50 [61.00, 91.00]	< 0.001		63.00 [54.00, 74.00]	84.00 [65.00, 96.00]	< 0.001
TB (µmol/L) (median [IQR])		23.20 [15.70, 37.20]	22.80 [14.85, 35.25]	0.493		20.90 [14.00, 30.10]	16.70 [13.00, 26.40]	0.405
DB (µmol/L) (median [IQR])		8.20 [4.80, 16.40]	6.30 [4.47, 18.33]	0.561		7.20 [4.00, 13.05]	6.30 [4.10, 9.50]	0.906
Urea (mmol/L) (median [IQR])		4.60 [3.80, 5.70]	5.35 [4.40, 6.78]	0.001		4.50 [3.60, 6.05]	5.60 [4.00, 7.10]	0.01
LDH (U/L) (median [IQR])		227.00 [182.00, 319.00]	258.00 [193.75, 418.75]	0.051		230.00 [182.50, 312.00]	302.00 [213.00, 462.00]	0.023
Ca^2+^(mmol/L) (median [IQR])		2.25 [2.13, 2.37]	2.18 [2.04, 2.30]	0.028		2.24 [2.14, 2.35]	2.28 [2.19, 2.37]	0.122
TG (mmol/L) (median [IQR])		1.48 [0.89, 3.58]	1.72 [1.00, 9.36]	0.105		1.33 [0.89, 2.96]	1.78 [0.94, 3.87]	0.242
GLU (mmol/L) (median [IQR])		7.54 [6.20, 9.65]	7.91 [6.32, 11.01]	0.326		7.20 [6.43, 10.13]	7.63 [6.94, 8.94]	0.635
ALT (U/L) (median [IQR])		56.00 [22.00, 192.00]	36.50 [20.00, 96.25]	0.122		39.00 [21.00, 179.50]	45.00 [25.00, 122.00]	0.63
AST (U/L) (median [IQR])		49.00 [22.00, 169.00]	34.50 [23.00, 91.50]	0.435		31.00 [18.00, 102.50]	42.00 [27.00, 101.00]	0.154
GGT (U/L) (median [IQR])		126.00 [40.00, 345.00]	134.50 [34.50, 300.25]	0.685		80.00 [33.00, 245.00]	71.00 [46.00, 233.00]	0.908
ALP (U/L) (median [IQR])		107.00 [80.00, 163.00]	101.00 [81.00, 146.25]	0.818		107.00 [83.50, 145.00]	102.00 [79.00, 161.00]	0.934
ALB (g/L) (median [IQR])		39.00 [35.50, 42.40]	36.30 [32.88, 40.02]	< 0.001		38.90 [35.75, 40.90]	37.60 [32.20, 40.20]	0.153
Na^+^(mmol/L) (median [IQR])		139.30 [137.30, 141.60]	138.10 [134.88, 140.85]	0.011		140.10 [136.15, 142.40]	138.40 [136.00, 140.20]	0.083
AMY(U/L) (median [IQR])		460.00 [145.00, 1415.00]	330.00 [97.25, 1162.50]	0.22		496.00 [200.50, 1416.00]	278.00 [104.00, 753.00]	0.268
K^+^(U/L) (median [IQR])		3.92 [3.63, 4.17]	3.78 [3.49, 4.10]	0.103		3.92 [3.71, 4.18]	4.01 [3.79, 4.49]	0.082
PT (s) (median [IQR])		13.40 [12.40, 14.60]	12.95 [11.97, 14.20]	0.132		13.40 [12.30, 14.55]	13.20 [12.30, 14.70]	0.839
INR (median [IQR])		1.09 [1.00, 1.19]	1.04 [0.95, 1.14]	0.018		1.11 [1.01, 1.20]	1.05 [0.98, 1.23]	0.277
APTT (s) (median [IQR])		32.60 [29.40, 37.30]	31.75 [28.70, 36.42]	0.34		31.90 [28.80, 34.95]	33.30 [29.40, 35.70]	0.446
CRP (mg/L) (median [IQR])		10.50 [2.30, 52.60]	12.35 [4.18, 96.70]	0.057		9.10 [2.00, 68.15]	6.00 [2.10, 54.00]	0.495

SBP: systolic blood pressure; DBP: diastolic blood pressure; MAP: mean artery pressure; N: neutrophil count; L: lymphocyte count; Lr: percentage of lymphocytes; Cr: creatinine; TB: total bilirubin; DB: direct bilirubin; TG: total triglycerides; GLU: glucose; ALB: albumin

The follow-up time for our patients was equivalent to their length of hospital stay, ranging from 1 to 37 days (with an average of approximately 10.05 days). According to the KDIGO guidelines, 97 patients (22.2%) developed AKI during the follow-up period. Among the cases of AKI, 77cases (79.4%) were of the least severe manifestation, while stage 2 AKI was observed in 16 cases (16.5%), and stage 3 in 4 cases (4.1%). Additionally, 8 patients (8.3%) received Continuous Renal Replacement Therapy (CRRT). The onset of AKI in our patients occurred between 1 and 21 days (with an average of approximately 4.74 days). Among them, 17 cases (17.5%) were diagnosed on the first day of admission. To perform internal validation, all patients were randomly divided into a training set (*n* = 313, 70%) and a validation set (*n* = 124, 30%) using randomization software. In the training and validation sets, 68 patients (21.7%) and 29 patients (23.4%), respectively, developed AKI. Additionally, there were a total of 2 deaths (0.5%) among all patients, both of whom had AKI, and 1 patient (0.2%) had an unknow outcome.

### Development of prediction model

**Fig. 2:**
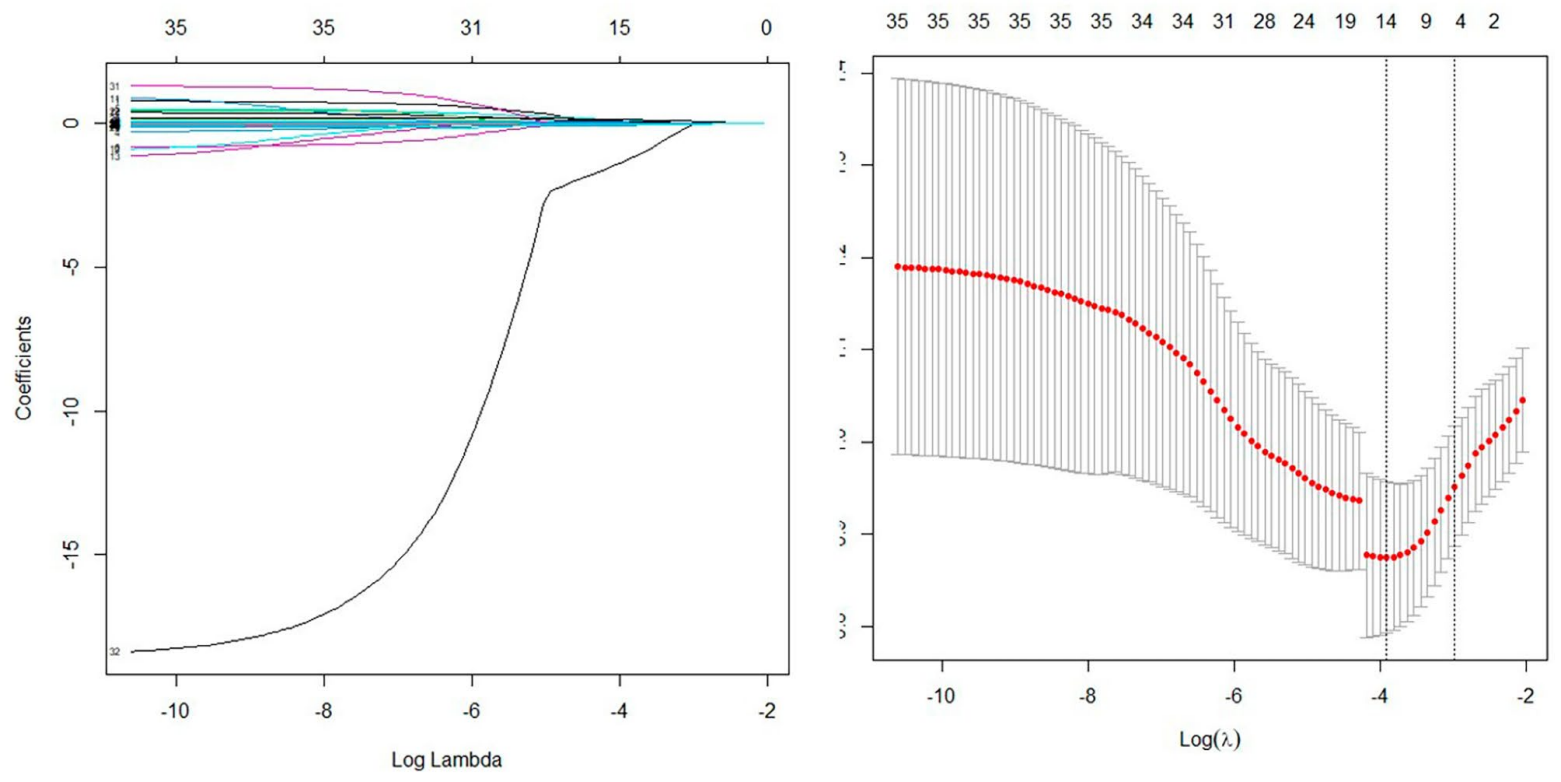
Penalty chart of predictive factors for acute kidney injury based on LASSO regression analysis

**Fig. 3:**
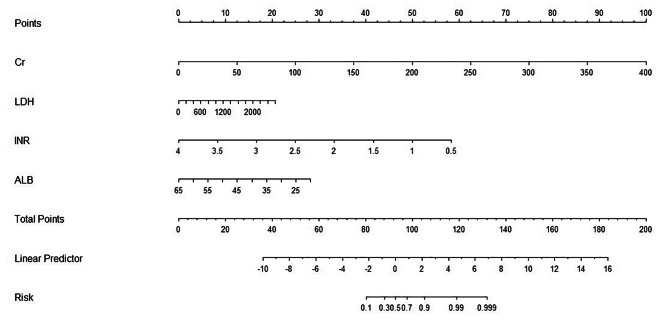
Nomogram of the LASSO model for the early prediction of acute kidney injury

**Fig. 4:**
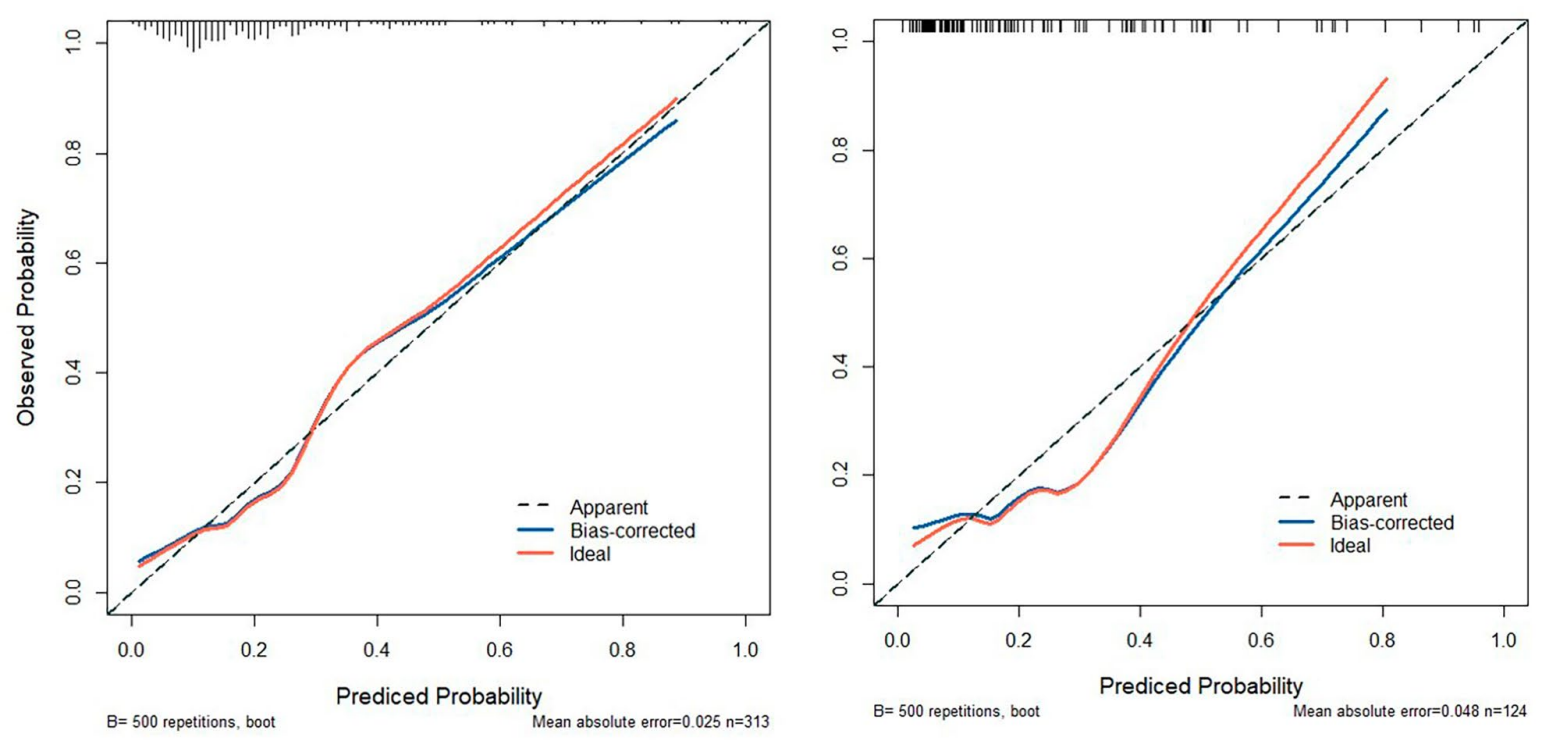
Calibration curve of the LASSO model in the training and validation set

**Fig. 5:**
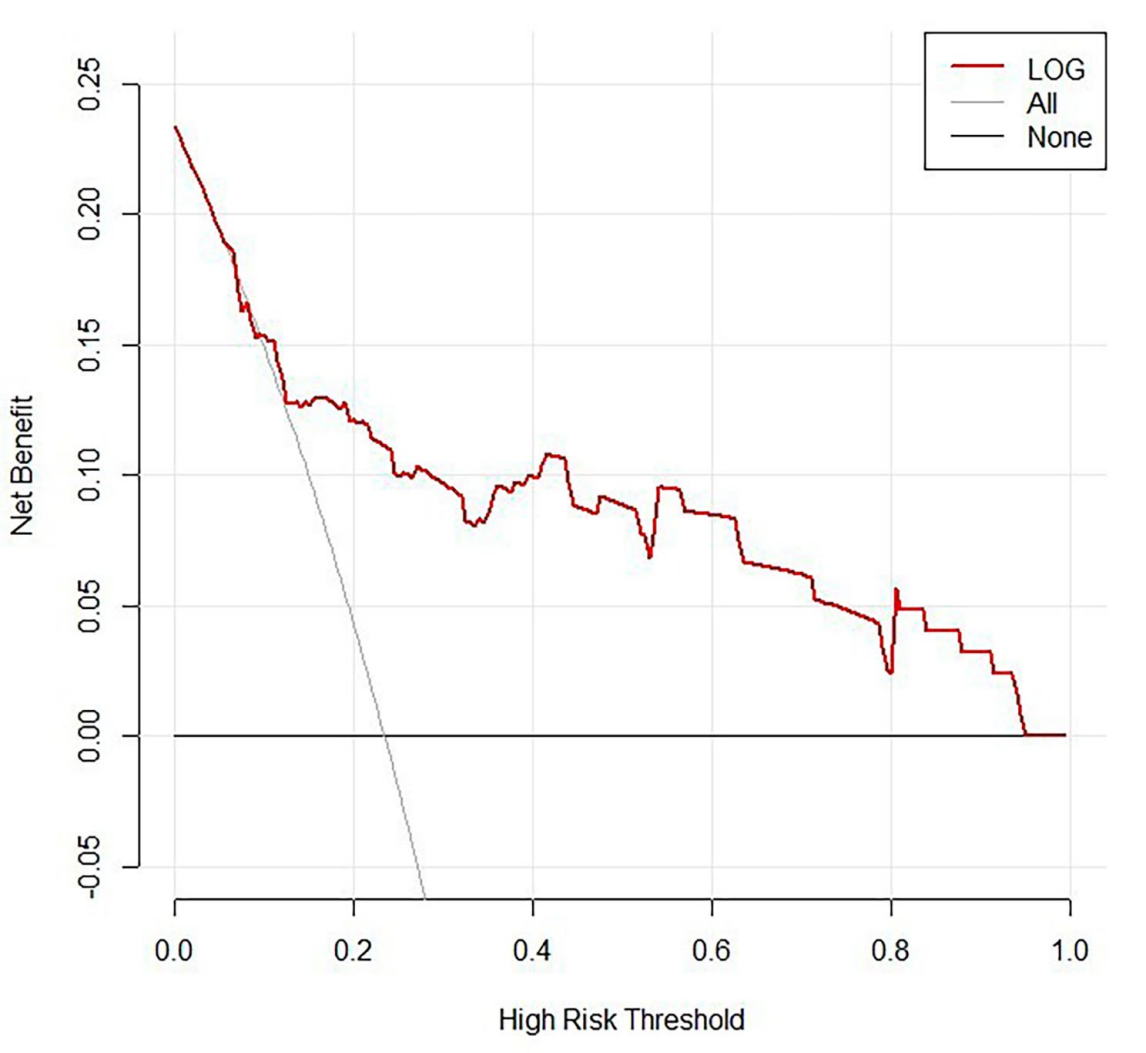
Decision curve analysis of the LASSO model in validation set

Univariate analysis was performed using LASSO regression with the “λmin (0.020)” criterion, and 5-fold cross-validation was used to solve the problem of multicollinearity among the variables (Fig. 2). Multivariate analysis was performed using stepwise LR, and four variables were identified as independent risk factors from 36 variables, and a nomogram was plotted (Fig. 3). The calibration curves of the training and validation sets are shown in Fig. 4, with mean absolute errors of 0.025 and 0.048, respectively. This indicates a high degree of reliability of the LASSO model in estimating risk compared to the observed risk. The DCA of the validation set indicated that within the predicted AKI probability threshold range of 20–90% by the LASSO model, an intervention could potentially provide additional benefits in the range of 1–12%. If a clinician believed there was a 40% likelihood of a patient would develop AKI, the DCA analysis from the validation set suggested that the patient could potentially benefit by 10% from early intervention. This would be equivalent to identifying 10 AKI patients and advising against any unnecessary treatment for every 100 patients. This constitutes a straightforward comparison with the “treat none” strategy (represented by the horizontal line in Fig. 5), which inherently results in zero true positives and zero false positives [24]. The net benefit indicates that implementing the LASSO model would enhance patient outcomes, without regard to the preferences of either the patient or the doctor. The ROC curve for the validation set is displayed in Supplementary Figure S1, with an AUC value of 0.799, as depicted in Table 2.

**Table 2:** Comparison of LR and AutoML models for early prediction of AKI in the validation cohort

AUC(95%CI)		***p***-value	Sensitivity	Specificity	Accuracy	PPV	NPV	LR+	LR−
**AutoML**									
**GBM**	0.812(0.705–0.801)	＜0.001	0.759	0.811	0.798	0.550	0.917	4.004	0.298
**DRF**	0.800(0.696–0.904)	＜0.001	0.655	0.853	0.806	0.576	0.890	4.446	0.404
**GLM**	0.734(0.630–0.838)	＜0.001	0.655	0.768	0.742	0.463	0.880	2.829	0.449
**DL** **Logistic regression**	0.830(0.734–0.926)	＜0.001	0.759	0.832	0.815	0.579	0.919	4.504	0.290
**LASSO**	0.799(0.694–0.905)	＜0.001	0.586	0.947	0.799	0.773	0.882	11.138	0.437

LR: logistic regression; AutoML: automated machine learning; AKI: acute kidney injury; PPV: positive predictive value; NPV: negative predictive value; LR+: positive likelihood ration; LR−: negative likelihood ratio

**Fig. 6:**
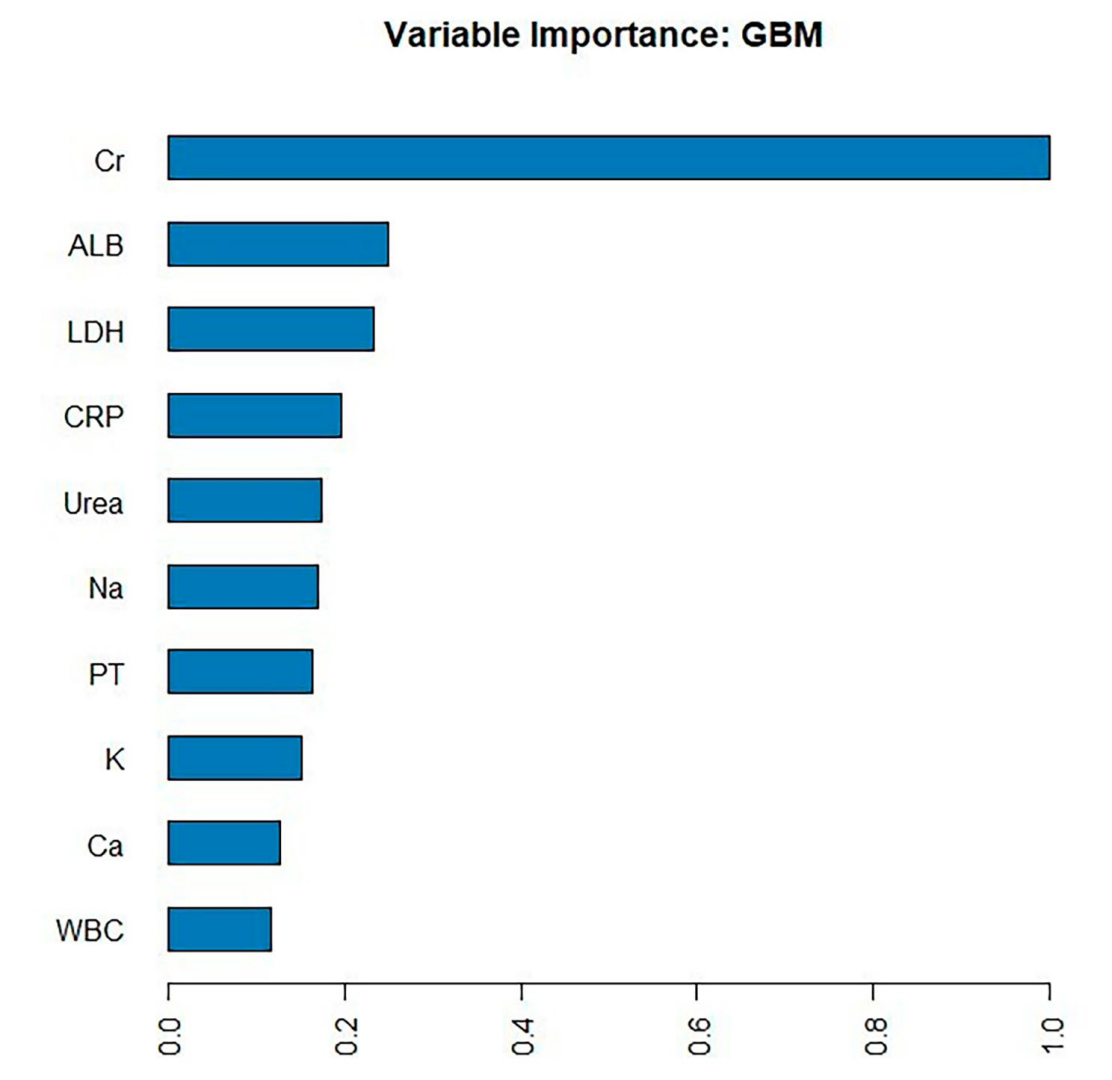
Variable importance of the GBM model in the training set

**Fig. 7:**
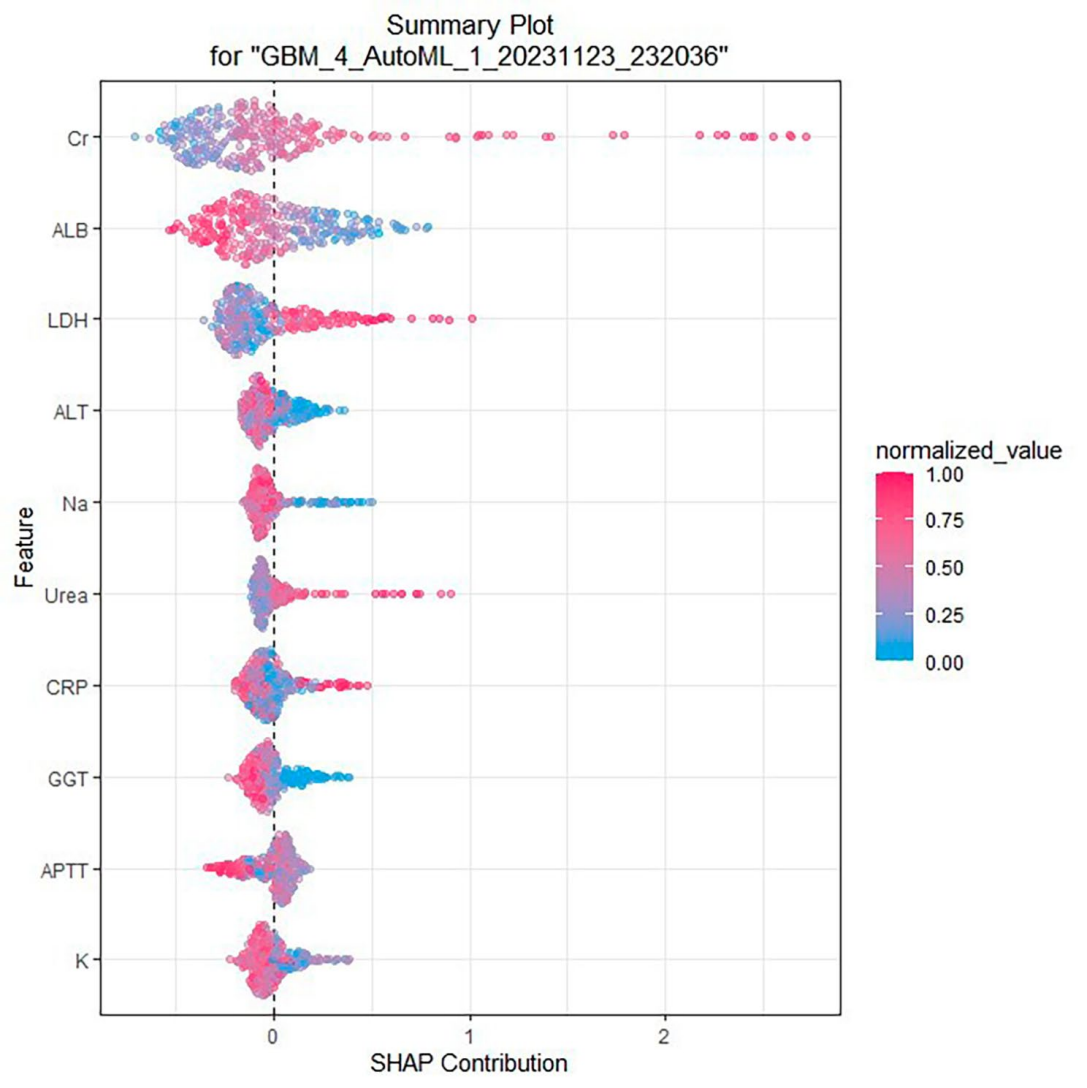
SHAP of the GBM model in the training set

We developed a total of 65 models using four machine learning algorithms (DL, GBM, GLM, and DRF) and excluded stacked ensemble models due to their poor interpretability. The DL model outperformed the other models, primarily because it achieved the highest AUC of 0.830 in the validation cohort, which was a comprehensive evaluation for imbalanced samples. As shown in Fig. 6, in the GBM model, creatinine (Cr), albumin (ALB) and lactate dehydrogenase (LDH) were the three most important features, followed by C-reactive protein(CRP)., blood urea nitrogen (Urea), sodium (Na^+^), prothrombin time (PT), serum potassium(K^+^ ), serum calcium(Ca^2+^)and white blood cell count(WBC). Additionally, Cr, LDH, and ALB were significant variables shared between the GBM model and the LASSO model. As shown in Fig. 7, in the SHapley Additive exPlanations (SHAP) plot of the GBM model, creatinine (Cr), albumin (ALB), lactate dehydrogenase (LDH), alanine aminotransferase(ALT), sodium (Na+), urea(Urea), C-reactive protein(CRP), gamma-glutamyl transpeptidase(GGT), activated partial thromboplastin time(APTT), and serum potassium(K^+^ ) were the top ten important variables. Variables with values approaching 1 exhibited a stronger correlation with an elevated risk of AKI progression in patients. For instance, the red segment of LDH, which was predominantly located to the right of the axis at 0, indicated that higher levels of LDH in AP patients were associated with an increased likelihood of developing AKI.

**Fig. 8:**
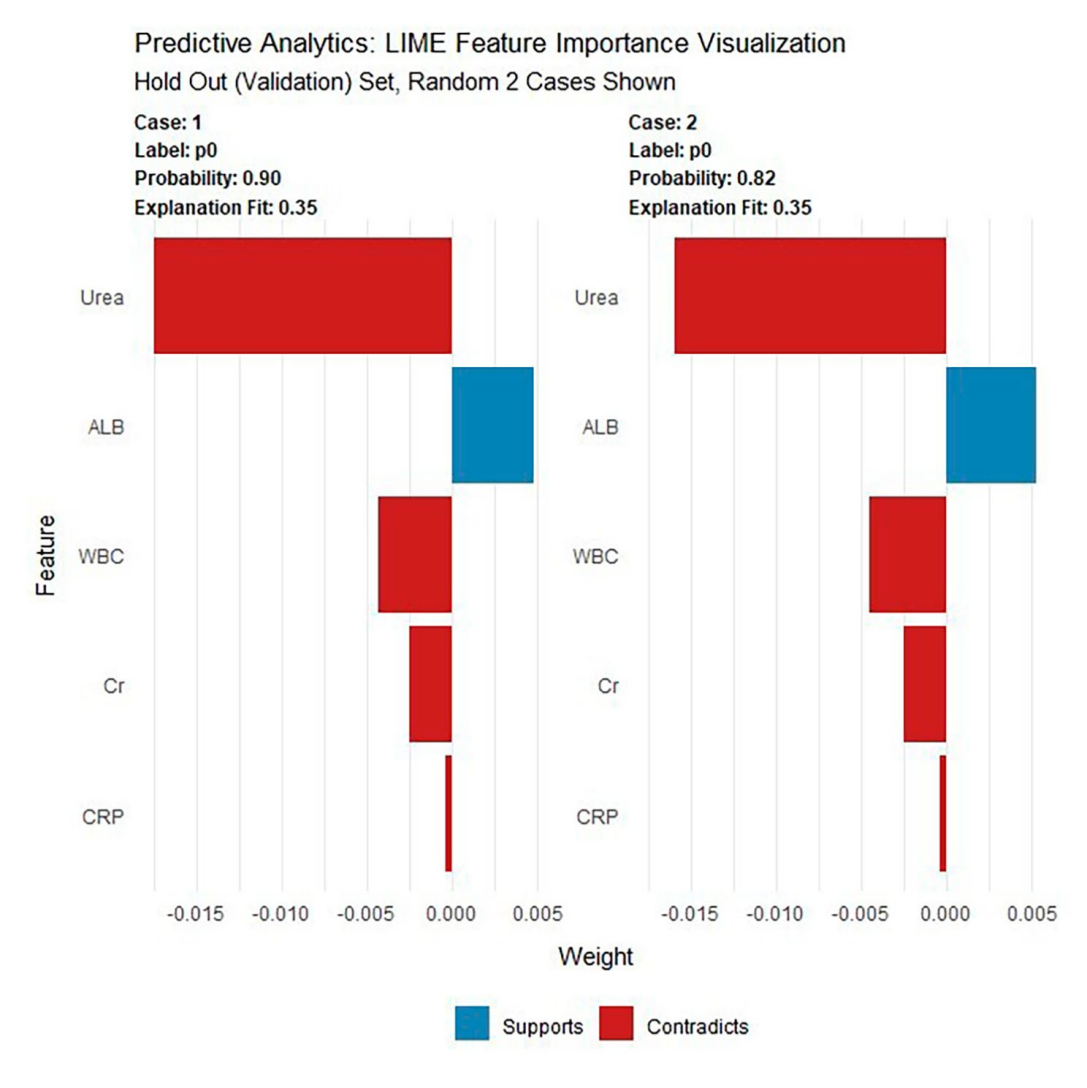
LIME of the DRF model in the validation set

The locally interpretable model-agnostic explanations (LIME) plot of the DRF model on the validation set demonstrated the contributions of several important variables to the development of AKI. As shown in Fig. 8, the DRF model predicts that both case 1 and case 2 have a high probability of developing AKI, with probabilities exceeding 0.80. In both cases, albumin (ALB) was a supportive feature for the development of AKI, while urea (Urea), white blood cell count (WBC), creatinine (Cr), and C-reactive protein (CRP) did not support its occurrence. The DCA results for the validation set demonstrated that if the threshold probability of AKI predicted by the AutoML models fell within the range of 10–100%, an intervention could potentially yield an additional benefit in the range of approximately 1–15%. According to the DCA of the validation set, when a clinician considered the patient had a 10% chance of developing AKI, the patient might gain at least about 15% of the benefit from an early intervention. This is a direct comparison with treat none (the horizontal line in Supplementary Figure S2), which has zero true positives and zero false positives by default.

### Comparisons models developed by LR and AutoML

In the validation set, the AUC values of five models were as follows: DL had the highest AUC of 0.830, followed by GBM with 0.812, DRF with 0.800, LASSO with 0.799, and GLM with 0.734. Among these models, the DL model had the highest AUC and accuracy, both exceeding 0.80, and ranked first. And the AUC of machine learning models were consistently higher than that of the LR model, which is a comprehensive evaluation index for model performance. Details are listed in Table 2.

## Discussion

AKI has been widely recognized as a major contributor to adverse outcomes in AP patients, with the ability to manifest at any point during the disease progression, thus exerting a significant impact on prognosis. Extensive research has revealed a substantial correlation between the occurrence of acute kidney injury and heightened risks of chronic kidney disease, myocardial infarction, stroke, and one-year mortality [25]. In this context, early and accurate prediction of which patients are more likely to develop AKI becomes crucial for proactive prevention and treatment.

In this research, we developed and validated a set of models using AutoML and LR, respectively, to enable early prediction of AKI. Compared to conventional univariate and sequential multivariate analyses, AutoML required less time and achieved higher accuracy, significantly enhancing work efficiency. Furthermore, the ensemble model integrated diverse ML algorithms and employed multiple-classifiers to predict the target outcome through a voting mechanism, thereby enhancing overall performance [26]. We employed four AutoML algorithms (GBM, DRF, GLM, and DL) to predict AKI early. All models outperformed traditional algorithm models, with the DL model ranking first in AUC on the validation set. AUC is a comprehensive indicator for evaluating model performance. As our goal is to early detect AP patients who may develop AKI, sensitivity is also important evaluation indicators. Both DL and GBM models have a sensitivity greater than 0.750. Therefore, in our study, the DL model performed the best.

In a research conducted in 2020, Wu et al. introduced a novel scoring system for forecasting organ failure in AP, which included LDH, creatinine, albumin, and serum calcium(Ca^2+^) as crucial variables [27]. LDH also played an important role in the classic RANSON score [28]. Several investigations have suggested the measurement of LDH activity (within 12 h of symptom onset) as a biomarker for early prognostic prediction in AP. These studies have disclosed that LDH activity can achieve a sensitivity of 63.6% and a specificity of 89.6% when discerning patients with distinct prognoses [29]. Yang et al. showed a significant correlation between elevated Cr and the high risk of AKI [19]. These findings are consistent with the results of this study, where LDH, creatinine and ALB were identified as important serum markers for early prediction of AKI in the GBM and LR models.

An increasing number of experimental and clinical studies have shown that the inflammatory response plays an indispensable role in the pathophysiology of AKI [29–31]. The inflammatory response has the potential to increase mucosal permeability, leading to the translocation of endotoxins. Endotoxins can promote the development of AKI by elevating endothelin levels, which in turn cause vasoconstriction, reduce renal blood flow, and result in tubular necrosis. Hence, systemic inflammatory markers like CRP and WBC may be useful in predicting AKI in AP patients, which is not surprising.

The levels of Urea can to a certain extent indicate volume depletion, kidney function, quality of resuscitation, and ischemic damage in patients with AP [32]. It has been reported that higher levels of Urea admission were associated with an increased risk of developing severe acute pancreatitis (SAP) [33]. Furthermore, Urea has been found to be a better predictor of persistent organ failure and pancreatic necrosis in acute pancreatitis compared to other laboratory indicators [34].

Multiple studies have indicated that imbalances in Na^+^ levels independently increase the risk of AKI [35, 36]. Given the kidneys’ pivotal role in maintaining the body’s fluid and electrolyte equilibrium, it’s not unexpected that renal dysfunction often correlates with disruptions in fluid balance and changes in serum electrolyte levels. Furthermore, research led by Lee et al. demonstrated that pre-existing hyponatremia elevates the probability of AKI occurrence in patients by approximately 30% [37].

PT representing the coagulation function of patients, was included in the list of the top 10 important features in the GBM model. This could be attributed to the fact that the hypercoagulable state of blood in patients with AP can contribute to tubular injury [38]. Moreover, hypocalcemia stands out as a key factor contributing to coagulation disturbances in individuals with AP [39]. On one hand, it is due to the release of pancreatic enzymes into the bloodstream, leading to the extensive breakdown of ALB and resulting in hypoalbuminemia, on the other hand, it is also due to the retention of calcium ions caused by the saponification of fatty acids released as a result of increased fat breakdown in abdominal adipose tissue [40]. Therefore, it is of great significance to strengthen the monitoring of Ca^2+^ and ALB in AP patients.

Lombardi G et al. demonstrated that deviations from normal potassium (K^+^) levels, as well as fluctuations within the normal range, were linked to the development of AKI. And, increased variability in K^+^ levels is independently associated with a higher risk of AKI, potentially due to its association with disturbances in acid-base balance [41].

The strength of this study lies in the use of AutoML to construct a series of models that can more accurately and sensitively predict AKI early compared to traditional algorithms. And the predictive factors in the models are routine detection indicators for AP, which have high clinical application value and are worth widespread application. However, this study also has some limitations. First, our analysis only used single-center data and included a relatively small number of patients. The performance of the predictive model may vary with data sets from other institutions with different patient characteristics. Second, we did not include novel biomarkers recently emphasized in research because they are not widely used in clinical practice. Third, we simply used serological indicators and did not incorporate variables such as body mass index (BMI), APACHE II, and IAP to comprehensively assess the risk of AKI in AP patients. This would affect the performance of our model. Fourth, our study is retrospective, and more prospective research is needed to further validate our findings.

## Conclusions

The models developed based on the AutoML platform can assess the risk of AKI in patients with AP early and accurately. Their performance surpasses that of scoring systems constructed using traditional algorithms, holding significant clinical value. This may provide a direction for the application of AutoML in future medical research.

### Electronic supplementary material

Below is the link to the electronic supplementary material.

Supplementary Material 1

Supplementary Material 2

Supplementary Material 3

## Data Availability

The datasets used and analysed during the current study are available from the corresponding author on reasonable request.
