# Supplementary material for: Automated machine learning for early prediction of acute kidney injury in acute pancreatitis
Source: BMC Med Inform Decis Mak. 2024 Jan 11;24:16. doi: 10.1186/s12911-024-02414-5 (PMC10785491; doi:10.1186/s12911-024-02414-5)
Supplement: Supplementary file 1 — Supplementary Material 1 [file 12911_2024_2414_MOESM1_ESM.doc]

Table S1. Attributes and variables extracted from electronic medical records.

| Attributes | Variables | group |
| --- | --- | --- |
| Demographic information | Sex (%) | male |
|  |  | female |
|  | Age (mean (median [IQR]) | |
|  | Smoke (%) | no |
|  |  | yes |
| Etiology | Etiology (%) | biliary |
|  |  | hyperlipidemia |
|  |  | alcoholic |
|  |  | others |
| Concomitant diseases | Hypertension (%) | no |
|  |  | yes |
|  | Diabetes (%) | no |
|  |  | yes |
| Hemodynamic parameters | SBP (median [IQR]) | |
|  | DBP (median [IQR])) | |
|  | MAP (median [IQR]) | |
| Laboratory tests | PLT (*10^9/L) (median [IQR]) | |
|  | WBC (*10^9/L) (median [IQR]) | |
|  | N (*10^9/L) (median [IQR]) | |
|  | L (*10^9/L) (median [IQR]) | |
|  | HCT (L/L) (median [IQR]) | |
|  | RDW (%) (median [IQR]) | |
|  | Lr (%) (median [IQR]) | |
|  | Cr (umol/L) (median [IQR]) | |
|  | TB (umol/L) (median [IQR]) | |
|  | DB (umol/L) (median [IQR]) | |
|  | Urea (mmol/L) (median [IQR]) | |
|  | LDH (U/L) (median [IQR]) | |
|  | Ca^2+^ (mmol/L) (median [IQR]) | |
|  | TG (mmol/L) (median [IQR]) | |
|  | GLU (mmol/L) (median [IQR]) | |
|  | ALT (U/L) (median [IQR]) | |
|  | AST (U/L) (median [IQR]) | |
|  | GGT (U/L) (median [IQR]) | |
|  | ALP (U/L) (median [IQR]) | |
|  | ALB (g/L) (median [IQR]) | |
|  | AMY (U/L) (median [IQR]) | |
|  | Na^+^(mmol/L) (median [IQR]) | |
|  | K^+^ (mmol/L) (median [IQR]) | |
|  | PT (s) (median [IQR]) | |
|  | INR (median [IQR]) | |
|  | APTT (s) (median [IQR]) | |
|  | CRP (median [IQR]) | |

SBP, systolic blood pressure; DBP, diastolic blood pressure; MAP, mean artery pressure; PLT, platelet count; WBC, white blood cell count; N, neutrophil count; L, lymphocyte count; HCT, hematocrit; RDW, red blood cell volume distribution width; Lr, percentage of lymphocytes; Cr, creatinine; TB, total bilirubin; DB, direct bilirubin; UREA ,urea; LDH, lactate dehydrogenase; Ca^2+^ ,serum calcium; TG, total triglycerides; GLU, glucose; ALT, alanine aminotransferase; AST, aspartate aminotransferase; GGT, gamma-glutamyl transpeptadase; ALP, alkaline phosphatase; ALB, albumin; AMY, amylase ; Na^+^,sodium; K ^+^, serum potassium; PT, prothrombintime; INR, international normalization ratio; APTT, activated partial thromboplastin time; CRP, C-reactive protein;
