# Supplementary material for: Automated machine learning for early prediction of acute kidney injury in acute pancreatitis
Source: BMC Med Inform Decis Mak. 2024 Jan 11;24:16. doi: 10.1186/s12911-024-02414-5 (PMC10785491; doi:10.1186/s12911-024-02414-5)
Supplement: Supplementary file 2 — Supplementary Material 2 [file 12911_2024_2414_MOESM2_ESM.docx]

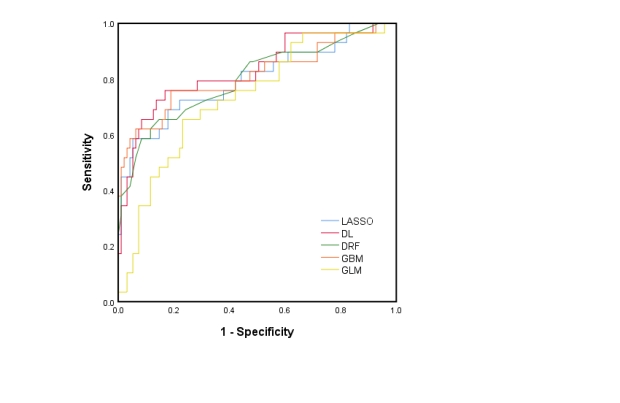


FigureS1 ROC curves of all proposed models (GBM, DL, DRF, GLM, and LASSO models) in the validation set. GBM= Gradient Boost Machine; DL= Deep Neural Nets; DRF= Default Random Forest; GLM= Generalized Linear Models; LASSO= the Least Absolute Shrinkage and Selection Operator;
