# Supplementary material for: Automated machine learning for early prediction of acute kidney injury in acute pancreatitis
Source: BMC Med Inform Decis Mak. 2024 Jan 11;24:16. doi: 10.1186/s12911-024-02414-5 (PMC10785491; doi:10.1186/s12911-024-02414-5)
Supplement: Supplementary file 3 — Supplementary Material 3 [file 12911_2024_2414_MOESM3_ESM.docx]

Figure S2 Decision curve analysis plots of the 4 models in the validation set, indicating net benefits of around 1%-15%. (A)GBM model; (B) DL model; (C) GLM model; (D) DRF model.


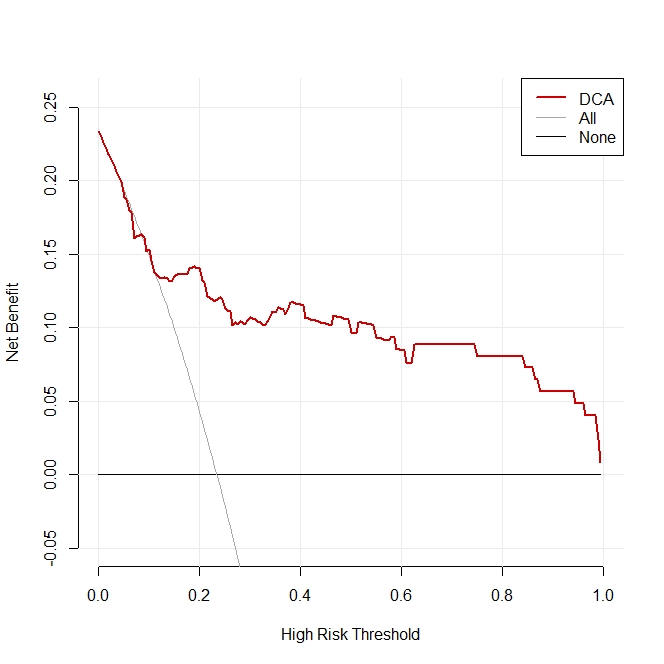

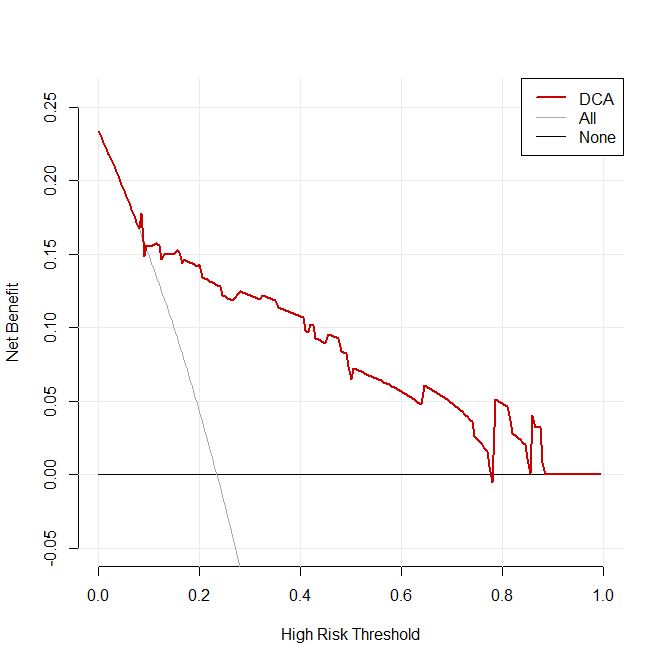

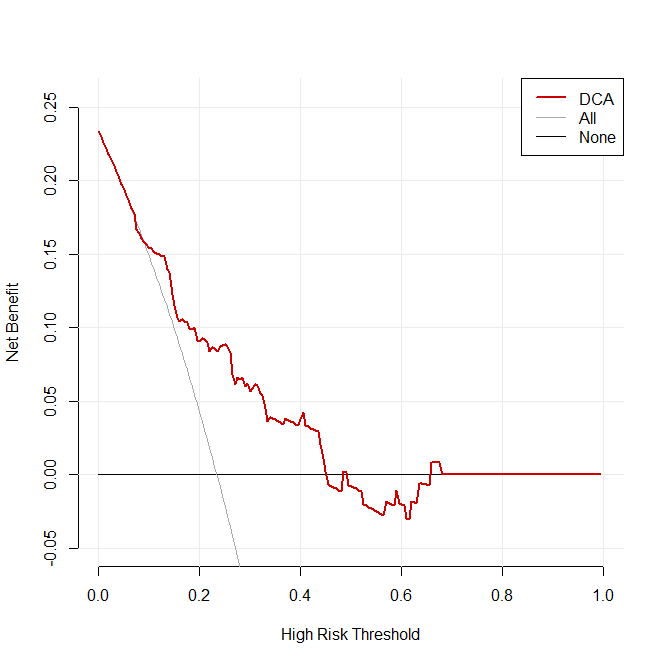

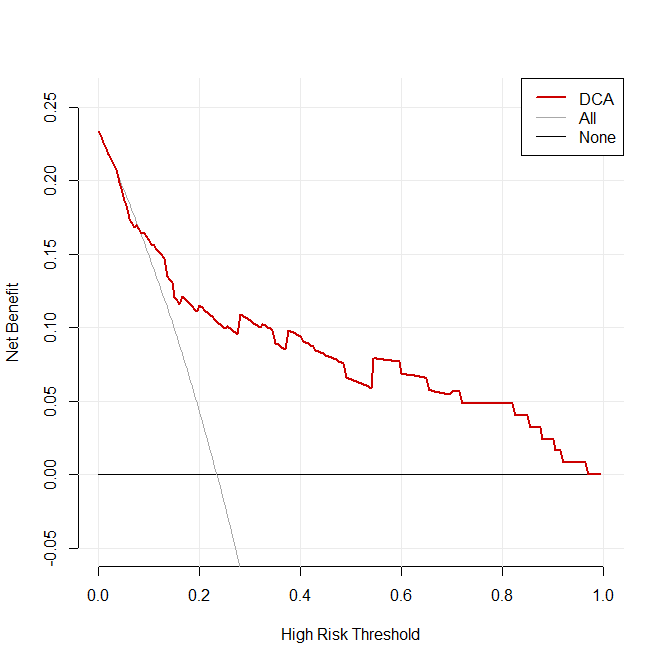


A

B

C

D
